# Direct Care Workers Employed as Medical Aides: Home Health Aides, Nursing Assistants, and Orderlies

**DOI:** 10.1093/geroni/igab046.3085

**Published:** 2021-12-17

**Authors:** Christopher Kelly, Jerome Deichert

**Affiliations:** University of Nebraska at Omaha, Omaha, Nebraska, United States

## Abstract

Purpose: This study describes the differences among direct workers (DCWs) employed as medical aides in three occupations: home health aides, nursing assistants, and orderlies. Design and Methods: Data were from the 1% Public Use Microdata Sample (PUMS) of the 2019 American Community Survey (ACS). Logistic regression was used to compare demographic and employment characteristics of DCWs employed as medical aides in three occupations: home health aides, nursing assistants and orderlies. Results: Compared to orderlies and psychiatric aides, home health aides are more likely to be foreign born, more likely to be female, less likely to work in institutional settings, less likely to be under age 25, less likely to work year-round full-time, less likely to have more education, and less likely to receive insurance from their employers. Implications: Since 2018, the PUMS of the ACS separates nursing, psychiatric, and home health aides (previously one occupational category) into three: home health aides, nursing assistants, and orderlies. This affords researchers a more precise understanding of this part of the direct care workforce. Home health aides represented more than 2/3 of DCWs employed as medical aides in 2019. Further, these workers are distinct among medical aides. More than nursing assistants and orderlies, home health aides are older, female, underinsured, foreign-born, and with limited education. This reflects both the barriers home health aides face to other occupations and also the preferences of their employers (which include private households). These findings have implications for the recruitment and retention of medical aides across all three occupations.

